# Mepolizumab treatment in a patient with previous liver transplantation: One year follow-up

**DOI:** 10.1016/j.rmcr.2023.101933

**Published:** 2023-10-16

**Authors:** Claudio Candia, Francesco Coppa, Lucia Abagnale, Francesco Perna, Antonio Molino

**Affiliations:** Respiratory Division, Department of Clinical Medicine and Surgery, University of Naples “Federico II”, Naples, Italy

**Keywords:** Mepolizumab, Severe asthma, Tacrolimus, Liver transplantation

## Abstract

Severe Asthma (SA) is characterized by inadequate disease control despite maximal inhalation therapy. In November 2021, a 68-years old female patient presented at our facility referring a worsening in her asthma-related symptoms and a high exacerbations rate. She reported a liver transplantation in 2015 and was in treatment with tacrolimus. We started treatment with Mepolizumab 100 mg once every 28 days and monitored her lung function as well as her lymphocytes subsets. After one year follow-up, the patient had a substantial improvement in lung function, exacerbation rate, daily OCS intake dose and no variation in the blood concentration of tacrolimus.

## 1 Introduction

According to the European Respiratory Society/American Thoracic Society definition, Severe Asthma (SA) is characterized by an inadequate control of the disease, despite the treatment with high-dose inhaled corticosteroids and additional controllers (long-acting inhaled beta 2 agonists, montelukast, and/or theophylline) or with oral corticosteroids (for at least six months per year) [1].

Mepolizumab is a monoclonal humanized antibody specifically designed to target Interleukin-5 (IL-5) in order to inhibit eosinophilic inflammation [2,3]. Mepolizumab is currently used in clinical practice to treat patients with eosinophilic severe asthma, thus leading to the reduction of disease exacerbations and the intake of oral corticosteroids (OCS) [[4], [5], [6]].

Tacrolimus is a mTOR inhibitor and is used as first-line therapy in liver transplantation in order to prevent allograft rejection and subsequent organ failure [7].

Several trials have been carried on so as to investigate Mepolizumab safety [[8], [9], [10]]; however, to our knowledge, there is no trial that assessed Mepolizumab's safety and efficacy in real-life post-transplantation immunosuppressed patients, as well as no case report of its usage in such kind of patients.

Therefore, the aim of the present case report is to describe our experience with Mepolizumab for treating severe asthma in a patient who had undergone a liver transplantation and was in treatment with tacrolimus.

## 2 Case report

I In November 2021, a 68-year-old Caucasian female with a history of type-2 Diabetes Mellitus, Bronchial Asthma, and liver transplantation came to our outpatient facility due to increased dyspnea and cough in the previous months.

She referred allergy to Artemisia spp. She denied any history of smoking and reported that she used to work as farmer in her youth.

The patient had developed a Liver Cirrhosis after contracting Hepatits C Virus during her youth, with subsequent Chronic Liver Failure – which had brought her to transplantation in 2015; the patient had been in therapy with tacrolimus since then.

The patient started experiencing asthma related symptoms during her fourth decade, and they had worsened in the last five years. She reported three acute exacerbations in the last twelve months and daily usage of prednisone 12,5 mg for over six months. She also exhibited two blood cell counts (September 2020 and May 2021), which showed absolute Eosinophil counts of 567 cells/mm3 and 623 cells/mm3 respectively.

At the physical examination the patient was slightly tachypneic at ease. Peripheral Oxygen Saturation was 95 %. At the auscultation of the thorax, expiratory wheezing and ronchi were present.

In the suspect of SA, we decided to admit her to Day Hospital in order to perform further evaluations.

At admission, we ran a complete panel of blood tests, parasitological stool analysis, a chest radiography, and a complete lung function assessment.

Laboratory data at admission are summarized in Table 1. In particular, she had an absolute Eosinophil count of 320 cells/mm3 and a Tacrolimus blood concentration of 3 ng/mL, which was within the reference values (Table 2).

**Table 1:** Domiciliary therapy at the first outpatient visit.

Drug	Posology
Tacrolimus	3 mg once daily
Pantoprazole	20 mg once daily
Bisoprolol	1,25 mg once daily
Tapazole	5 mg once daily
Acetylsalicylic acid	100 mg once daily
Insulin lispro	8 U twice daily
Insulin glargine	10 U once daily
Vitamin D	25,000 U once every two weeks
Budesonide/Formoterol	160/4,5 mcg two puffs twice daily
Tiotropium bromide	5 mcg once daily
Prednisone	12,5 mg once daily

**Table 2:** Main outcomes assessed during the follow-up.

	T0	T3	T6	T12
ACT	8	18	17	18
ACQ5	21	13	11	12
FEV_1_ (L)	1,20	1,32	1,33	1,33
FEV_1_ (%)	67,8 %	77,2 %	77,8 %	77,8 %
FVC (L)	2,13	2,15	2,16	2,14
FVC (%)	94,6 %	99,5 %	100 %	94,9 %
FEV_1_/FVC	56,3 %	61,4 %	61,6 %	61,5 %
RV (%)	91,6 %	92,3 %	93,8 %	93,6 %
TLC (%)	93,2 %	94,4 %	93,8 %	94,2 %
Tacrolimus (ng/mL)	3,2	2,9	3,7	3,5
CD3^+^	62,3 %	63,4 %	61 %	72,0 %
CD4^+^	44,5 %	46 %	42,3 %	52,0 %
CD8^+^	17,3 %	22,4 %	18,1 %	23,0 %
CD3^-^/CD56^+^	14,6 %	14,9 %	13,8 %	15,6 %
CD19^+^	22,5 %	23,8 %	26,3 %	25,0 %
CD4^+^/CD8^+^	2,6	2,2	2,4	2,3
CD25^+^	0	/	/	0
Eos (n)	320	57	83	40
Eos (%)	5,1 %	0,4 %	0,9 %	0,6 %

In order to exclude other underlying causes of blood eosinophilia, a parasitological stool analysis was performed on three different samples collected in three different days.

**Fig. 1:**
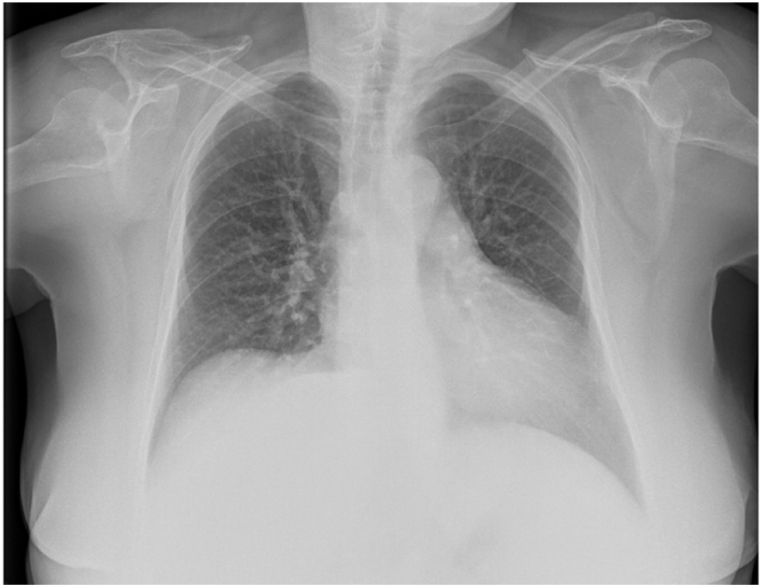
Evidence of peribronchial cuffing and hilar congestion at the Chest X-Ray.

Chest Radiograph showed hilar congestion and central peribronchial cuffing (Fig. 1).

At the spirometry a partially reversible obstructive syndrome was detected as well as a slight reduction in lung diffusion. No pathological findings were reported in the 6-min walking test and arterial blood gases. Fractional exhaled Nitric Oxyde (FeNO) resulted pathologically increased (reference value: <25 ppb).

Astma Control Test (ACT) and Asthma Control Questionnaire-5 (ACQ5) were also performed.

Lung Function Assessment values are reported in Table 2.

We confirmed our suspect of SA and considered treatment with Mepolizumab 100 mg. We also decided to run a Lymphocytes Subset Count before starting treatment, in order to verify whether it were affected by the administration of the drug. We decided to repeat the assessment at different timepoints, as shown in Table 2.

After the first injection, the patient reported no local side effects and was kept on observation for 2 h. She was discharged with the indication of administrating Mepolizumab every 28 days regularly and a tapering of her OCS intake until complete suspension three weeks later.

The patient entered a follow-up which consisted of new visits and lung function assessments at 3, 6 and 12 months.

At three months, the patient reported a significative improvement of the asthma-related symptoms. Moreover, there was an improvement in lung function assessments and asthma questionnaires.

We monitored the blood concentration of tacrolimus, which remained withing the range of safety and efficacy. Such results were still present at 6 and 12 months. No acute exacerbation was reported.

## 3 Discussion

Several trials have been carried on in order to demonstrate the safety and the efficacy of Mepolizumab in Severe Asthma, also in clinically severe conditions [11]. However, at a thorough literature research on PubMed, Embase and Scopus, no case report about the usage of Mepolizumab in Severe Asthma involving an immunosuppressed patient was found.

Our patient met the diagnostic criteria for Severe Asthma. In fact, despite the maximal standard of care inhalation therapy, she kept on experiencing acute exacerbations and needed almost continuous OCS treatment. The introduction of the biological drug allowed her to improve her lung function as well as her quality of life; most importantly, she reached a satisfactory disease control without the chronic administration of OCS. OCS in asthmatic patients have been linked to an increased risk of adverse effects (AE) as well as increased odds for osteoporosis, diabetes mellitus type 2 and obesity [12]. As a result, the patient lost weight (at 12 months: 3,5 kg, without changing eating habits) and reported a reduction in the sensation of hunger and nocturnal restlessness.

We monitored the blood concentration of tacrolimus and found it within normal range at every given timepoint. The patient underwent two hepatological and immunological assessments within the 12 months of follow-up, and no therapy was modified during such timeframe.

We also supposed that Mepolizumab could alter the distribution of the lymphocyte subsets, as tacrolimus has been known to do [13]. Therefore, we decided to monitor the lymphocyte subsets composition in order to understand whether Mepolizumab could have a role in a redistribution of the cell populations. Our patient had an increase of the B-cell aliquot (22,5 % vs normal range 10–15 %) which might be explained by the immunosuppression given by tacrolimus. B-cells, however, tended to be stable during the follow-up, with no significant variation between the different timepoints. We also tried to dose CD4^+^ CD25^+^ T regulatory cells (CD25 Tregs), which express IL-5 receptors on their surface and whose dysregulation has been linked to autoimmune diseases [14], with the hypothesis that Mepolizumab might induce a significative depletion in their amount. No significative variation was measured, although Tregs were evaluated only at the beginning of the treatment and at one-year follow-up, due to laboratory limitations.

## 4 Conclusion

Our case report showed that using Mepolizumab to treat Severe Asthma in a pharmacologically immunosuppressed patient has a detectable efficacy and has no effect on tacrolimus’ blood concentrations nor on lymphocyte subsets.

We therefore hope that clinicians who normally use such drug might be relieved when having to make the decision of starting the treatment in an immunosuppressed patient. In the end, the correct usage of the drug and a careful selection of eligible patients, may help obtain a better control of their disease and a subsequent improvement in their quality of life.

## Financial disclosure

None to declare.

## Authors’ contribution

All authors contributed equally to the manuscript and approved its final form

## Informed consent

The patient gave consent for the publication.

## Declaration of competing interest

The authors declare that they have no known competing financial interests or personal relationships that could have appeared to influence the work reported in this paper.
